# Pemphigus vulgaris as the first manifestation of multiple myeloma: a case report

**DOI:** 10.1186/s13256-018-1791-z

**Published:** 2018-09-07

**Authors:** Fandresena Arilala Sendrasoa, Irina Mamisoa Ranaivo, Mendrika Fifaliana Rakotoarisaona, Onivola Raharolahy, Naina Harinjara Razanakoto, Malalaniaina Andrianarison, Lala Soavina Ramarozatovo, Fahafahantsoa Rapelanoro Rabenja

**Affiliations:** Department of Dermatology, University Hospital Joseph Raseta Befelatanana, Antananarivo, Madagascar

**Keywords:** Pemphigus vulgaris, Multiple myeloma, Skin, Remission

## Abstract

**Background:**

The association between pemphigus and malignancy has been well documented for decades but an association between pemphigus vulgaris and multiple myeloma is unusual. We report a case of pemphigus vulgaris revealing multiple myeloma.

**Case presentation:**

A 55-year-old Malagasy man, with no significant past medical history, presented with bullous and erosive skin lesions involving his trunk and scalp for the last 2 months. He had no mucous membrane involvement. A diagnosis of pemphigus vulgaris was made on skin biopsy and direct immunofluorescence of perilesional skin revealing immunoglobulin G deposition in the intercellular spaces in the epidermis. In an enzyme-linked immunosorbent assay, his serum autoantibody index against desmoglein-1 and 3 was found to be 112 RU/mL and 34 RU/mL respectively. Serum immunoelectrophoresis showed a monoclonal gammopathy with a markedly elevated immunoglobulin G level (2880 mg/dL) in association with a lambda free light chain. Bone marrow aspirate showed 6% plasma cell infiltration. Further investigations, including creatinine blood test and whole body radiographic examinations, showed that he had initially clinical stage I multiple myeloma of the immunoglobulin G-λ type. Six months later, bone tomography revealed vertebral compression fractures of the thoracic and lumbar spine that correlated with his back pain topographically. Anti-myeloma treatment including melphalan and prednisone led to an immediate decline in monoclonal immunoglobulin G concentration. Skin and hematologic remission were maintained for 12 months.

**Conclusions:**

Absence of mucosal involvement, lack of vacuolar degeneration at the interface, and absence of apoptotic, dyskeratotic keratinocytes ruled out paraneoplastic pemphigus in our case. Pemphigus vulgaris should be considered even if possible underlying disease for which paraneoplastic pemphigus is recognized is present.

## Background

Pemphigus is a group of chronic blistering disorders of the skin and mucosal membranes caused by autoantibodies against epithelial cell adhesion molecules. The association between pemphigus and malignancy has been well known for decades [1] but the association between pemphigus vulgaris (PV) and multiple myeloma is unusual. We report a case of PV revealing multiple myeloma, in which the clinical features and results of immunoserological examinations were not characteristic of paraneoplastic pemphigus (PNP).

## Case presentation

**Fig. 1:**
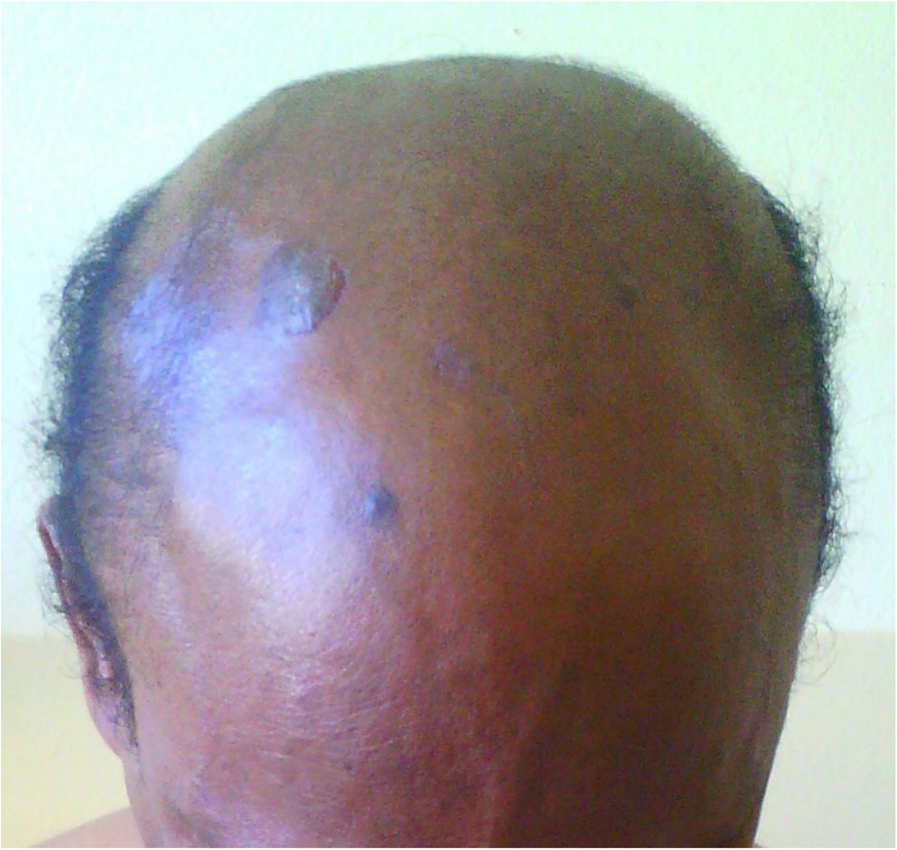
Multiple crusted erosions intermixed with erythematosus patches over scalp

**Fig. 2:**
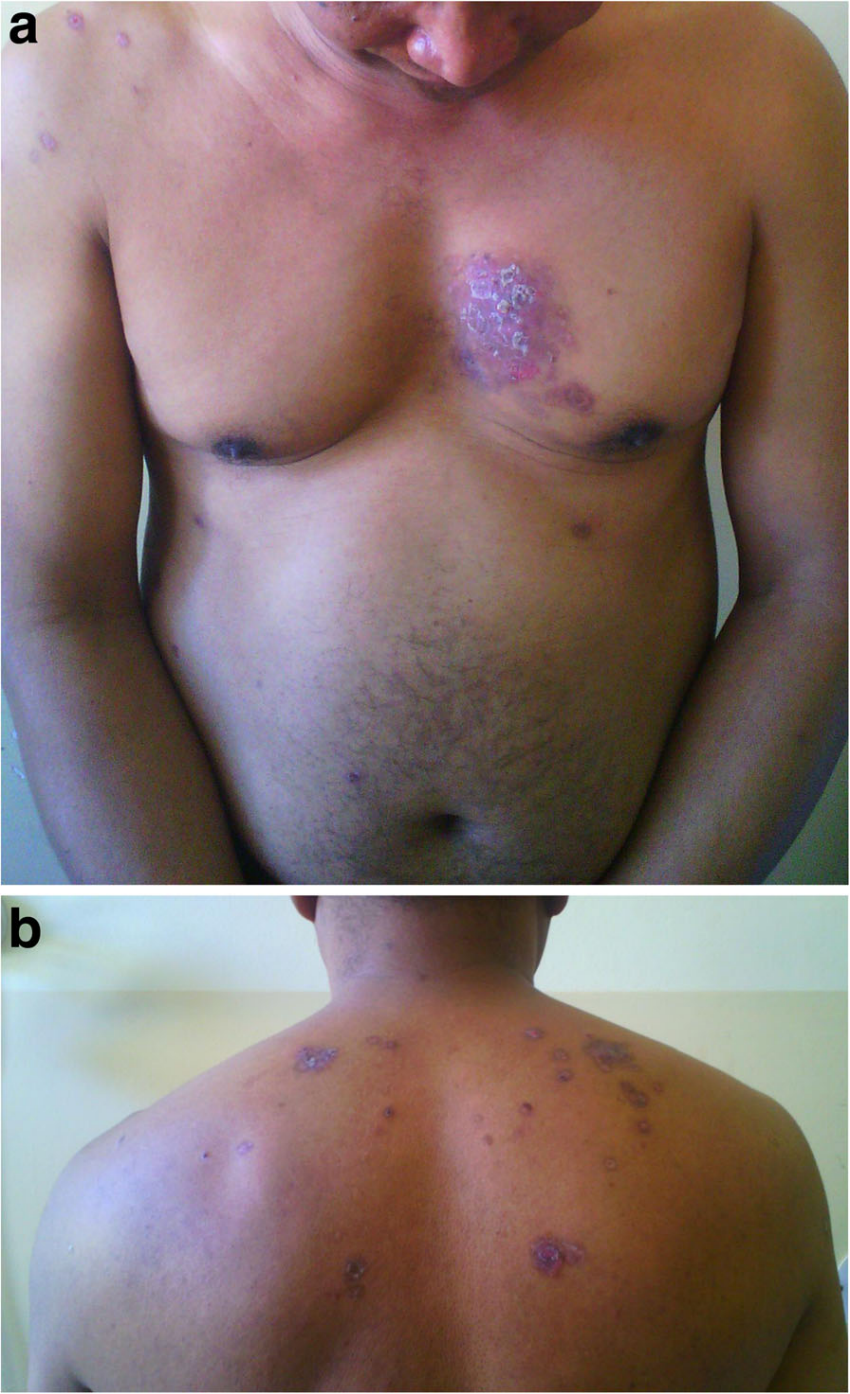
**a** Multiple crusted erosions intermixed with erythematosus patches over trunk. **b** Multiple crusted erosions intermixed with erythematosus patches over the back

A 55-year-old Malagasy man, a doctor, a non-smoker of tobacco, with no significant past medical history, presented with bullous and erosive skin lesions involving his trunk and scalp for the past 2 months. No toxic exposure was noted. He had a family history of cancer; his mother and sister presented breast cancer and multiple myeloma, respectively, the diagnosis of which were delayed. He had no personal or family history of any autoimmune disease. No medication was prescribed prior to diagnosis. A physical examination revealed multiple crusted erosions intermixed with erythematosus patches over his scalp (Fig. 1), trunk (Fig. 2a), and his back (Fig. 2b). He had no mucous membrane involvement. General physical and systemic examinations were normal.

A complete blood count revealed microcytosis without anemia with mean corpuscular volume (MCV) of 76 fl and hemoglobin of 15.7 g/dL; his white cell count and platelet count were normal. Alanine and aspartate aminotransferase were normal (28 U/L and 25 U/L, respectively) but serum creatinine was high (121 umol/l; normal range: 53–115 umol/L). Other laboratory tests including corrected calcium level, phosphoremia, lactate dehydrogenase, and urine analysis were normal. His HIV status was negative.

A skin biopsy showed suprabasal blisters containing eosinophils and acantholytic keratinocytes. Direct immunofluorescence of perilesional skin revealed immunoglobulin G (IgG) deposition in the intercellular spaces in the epidermis. In an enzyme-linked immunosorbent assay (ELISA), his serum autoantibody index against desmoglein-1 and 3 was found to be 112 RU/mL and 34 RU/mL (normal range, < 20 RU/mL), respectively. Serum immunoelectrophoresis showed a monoclonal gammopathy with a markedly elevated IgG level (2880 mg/dL) in association with a lambda free light chain. Urine analysis was negative for Bence-Jones protein and beta2-microglobulin was 2.4 mg/L. Bone marrow aspirate showed 6% plasma cell infiltration. Further investigations, including creatinine blood test and whole body radiographic examinations, showed that he had clinical stage I multiple myeloma of the IgG-λ type.

At first, the skin lesions regressed significantly after topical applications of corticosteroid ointment and no specific therapy for myeloma was conducted. Three months later, the dermal affliction occurred again so systemic administration of prednisolone (1 mg/kg per day) was started. Six months later, bone tomography revealed vertebral compression fractures of the thoracic and lumbar spine that correlated with our patient’s back pain topographically. However, his IgG level decreased (1080 mg/dL). Anti-myeloma treatment including melphalan and prednisone was started, which resulted in a rapid decline of monoclonal IgG concentration immediately. Skin and hematologic remission were maintained for 12 months.

## Discussion

Considering the results of routine microscopic sections, immunohistopathological examinations, and immunoserological analysis, our case definitely has PV but not PNP which is known to be associated with multiple myeloma. To the best of our knowledge, there have been no reported cases of multiple myeloma revealed by PV in Madagascar.

Skin lesions are frequently encountered in clinical practice; they can be a presentation of systemic diseases not excluding an occult malignancy. The association of pemphigus with malignancy has been well documented [2, 3]. PNP, a new disease entity, was first described by Anhalt *et al.* in 1990 [4]; PNP is associated primarily with malignant and benign tumors of hematological tissue origin. However, PV associated with multiple myeloma is rare. Kridin *et al*. reported 16 cases of multiple myeloma in 1985 patients with pemphigus [5]. Absence of mucosal involvement, lack of vacuolar degeneration at the interface, and absence of apoptotic, dyskeratotic keratinocytes ruled out PNP in our case. Furthermore, our patient’s skin lesion was not as severe or as polymorphous as those in PNP. PV and PNP have similar clinical presentations but have different pathophysiologies in that PV shows a more favorable prognosis than PNP, which often leads to pulmonary involvement. PNP is known to associate with diverse conditions such as non-Hodgkin’s lymphoma, chronic lymphocytic leukemia, Castleman’s disease, thymoma, and Waldenstrom’s macroglobulinemia; however, PV should be considered even if the possible underlying diseases for which PNP is recognized are present. Table 1 shows salient features to differentiate between PV and PNP.

**Table 1:** Salient features to differentiate between pemphigus vulgaris and paraneoplastic pemphigus

	Pemphigus vulgaris	Pemphigus paraneoplastic
Clinical findings	• Oral erosions particularly on the labial and buccal mucosa	• Painful, progressive stomatitis involving the tongue • Blisters and targetoid lesions on the palms and soles
Direct immunofluorescence	• Intercellular deposition of IgG and C3 in “chicken-wire” lattice pattern	• IgG deposition in all layers of the epidermidis and C3 in the lower epidermis and basement membrane • Intercellular staining is focal and faint
Autoantibodies	• Autoantibodies against desmoglein-1 and desmoglein-3	• Autoantibodies against desmoglein-1 and desmoglein-3 • Autoantibodies against proteins in the plakin family (plectin, desmoplakin I, desmoplakin II, bullous pemphigoid antigen I, envoplakin, and periplakin)
Visceral involvement	• Very rare	• Mucous membranes of the esophagus, stomach, duodenum, intestines, and pulmonary epithelium

*IgG* immunoglobulin G

Scleromyxedema, lichen myxedematosus, and papular mucinosis are the skin disorders most associated with monoclonal gammopathy [6, 7]. PV concomitant with multiple myeloma was first reported by Motoki Kurokawa *et al*. in 2005 [8]. Furthermore, there are several reports of cases of myeloma associated with autoimmune diseases such as autoimmune hemolytic anemia [9], acquired von Willebrand disease [10], Sjögren’s syndrome [11], and Hashimoto’s thyroiditis [12]. Although multiple myeloma is basically a B cell malignancy, the mechanism of autoantibody production in myeloma is still unclear. Fas mutations have been found in multiple myeloma, and there is a high incidence of autoreactive phenomena in patients with Fas mutations, such as systemic lupus erythematosus, Sjögren’s syndrome, and Hashimoto’s thyroiditis.

## Conclusions

Our patient’s case illustrates an uncommon occurrence of PV and multiple myeloma, presumably the first reported case in Madagascar. PV should be considered even if possible underlying disease for which PNP is recognized is present.

## Abbreviations

ELISA: Enzyme-linked immunosorbent assay
MCV: Mean corpuscular volume
PNP: Paraneoplastic pemphigus
PV: Pemphigus vulgaris
